# Kartagener’s syndrome: a case report

**DOI:** 10.1186/s13256-017-1538-2

**Published:** 2018-01-10

**Authors:** Abilo Tadesse, Hailemariam Alemu, Mezgebu Silamsaw, Yonathan Gebrewold

**Affiliations:** 1 Department of Internal Medicine, College of Medicine and Health Sciences, University of Gondar, Gondar, Ethiopia; 2 Department of Radiology, College of Medicine and Health Sciences, University of Gondar, Gondar, Ethiopia

**Keywords:** Kartagener’s syndrome, Primary ciliary dyskinesia, Chronic sinusitis, Bronchiectasis, Situs inversus

## Abstract

**Background:**

Kartagener’s syndrome is a subset of primary ciliary dyskinesia, an autosomal recessive inherited disorder characterized by the clinical triad of chronic sinusitis, bronchiectasis, and situs inversus. Abnormal ciliary structure or function leading to impaired ciliary motility is the main pathophysiologic problem in Kartagener’s syndrome.

**Case presentation:**

A 24-year-old man from Gondar town, North-West Ethiopia, presented to University of Gondar Hospital with recurrent episodes of nasal congestion with itching and paranasal discomfort, and productive cough for more than a decade. Clinical and imaging findings revealed chronic sinusitis, bronchiectasis, dextrocardia, and situs inversus. He was treated with orally administered antibiotics, mucolytic, and chest physiotherapy. He was symptomatically better with the above therapy, and started on a long-term low-dose prophylactic antibiotic.

**Conclusions:**

Patients with Kartagener’s syndrome exist in Ethiopia as cases of chronic recurrent sinopulmonary infections. As there is no easy, reliable non-invasive diagnostic test for Kartagener’s syndrome and the correct diagnosis is often delayed by years, it may cause chronic respiratory problems with reduced quality of life. Genetic counseling and fertility issues should be addressed once Kartagener’s syndrome is diagnosed.

## Background

Kartagener’s syndrome (KS) is a rare autosomal recessive genetic disorder which was first described by Siewert in 1904; however, Kartagener recognized the clinical syndrome in 1933. The syndrome includes the clinical triad of chronic sinusitis, bronchiectasis, and situs inversus [1, 2]. Camner *et al.* first suggested ciliary dyskinesia as the cause of KS in 1975. In 1977, Eliasson *et al*. first coined the term “immotile cilia syndrome” for KS to categorize infertility with chronic sinopulmonary infections [3, 4].

Normal ciliary function is critical for respiratory host defense and motility of sperm, and ensures proper visceral orientation during embryogenesis. In KS, the gene mutation at *DNAI1* and *DNAH5* leads to impaired ciliary motility, which predisposes to recurrent sinopulmonary infections, infertility, and errors with left–right body orientation [5, 6]. We report the case of a 24-year-old man with KS.

## Case presentation

A 24-year-old man from Gondar town, North-West Ethiopia, presented to our medical out-patient clinic, University of Gondar Hospital in first week of June 2017. He presented with the chief complaint of recurrent episodes of nasal congestion with itching and paranasal discomfort, and productive cough for more than a decade. He had repeated clinic visits since then, and had been treated as having chronic sinusitis and recurrent pneumonia. He noticed frequent exacerbation of cough with copious purulent sputum in the last 3 years. He was treated for pulmonary tuberculosis 7 years back as smear-negative pulmonary tuberculosis, but there was no significant clinical improvement after completion of 6 months’ anti-tuberculosis therapy. He was seen by an ear, nose, and throat (ENT) specialist 3 months back and was told he had chronic sinusitis and nasal polyp, and was treated with antibiotics and intranasal steroid. He was a casual alcohol consumer, but never smoked cigarettes. There was no similar illness in his family.

On physical examination, he was nourished, conscious, and oriented. His blood pressure (BP) was 100/70 mmHg, pulse rate (PR) 90 beats per minute, respiratory rate (RR) 20 breaths per minute, and temperature (T°) 37.5 °C. His arterial oxygen saturation (SaO_2_) was 93% with room air. He had hyperemic conjunctivae. He had a deviated left nasal septum with 1 × 2 cm-sized nasal polyp, and hypertrophied inferior turbinate. There was no lymphadenopathy in accessible sites. A respiratory system examination revealed coarse crackles and scattered rhonchi on both basal lung fields. On cardiovascular examination, apex beat was felt on right fifth intercostal space along midclavicular line. Heart sounds were best audible on the right side of his chest. An abdominal examination revealed tympanitic note on percussion and no sign of fluid collection. He had grade 2 clubbing of fingers of both hands. A nervous system examination showed no abnormality.

**Fig. 1:**
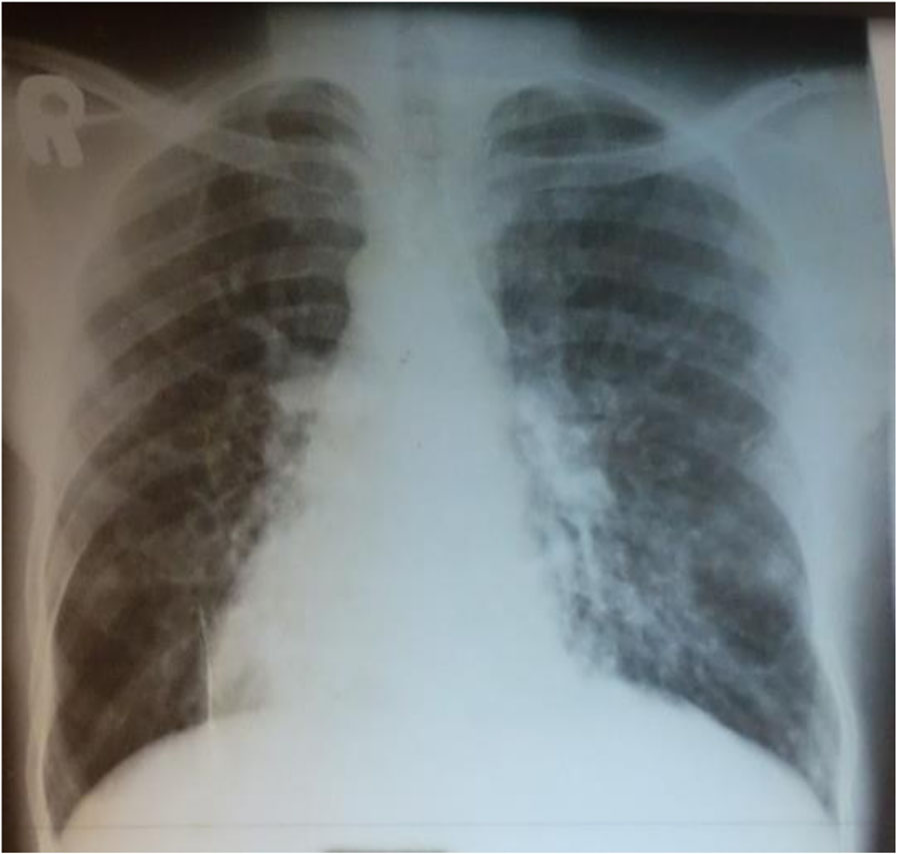
Posteroanterior chest X-ray showing dextrocardia with right-sided aortic arch. There is paracardiac and left lower lung bronchiectasis with fibrotic bands

**Fig. 2:**
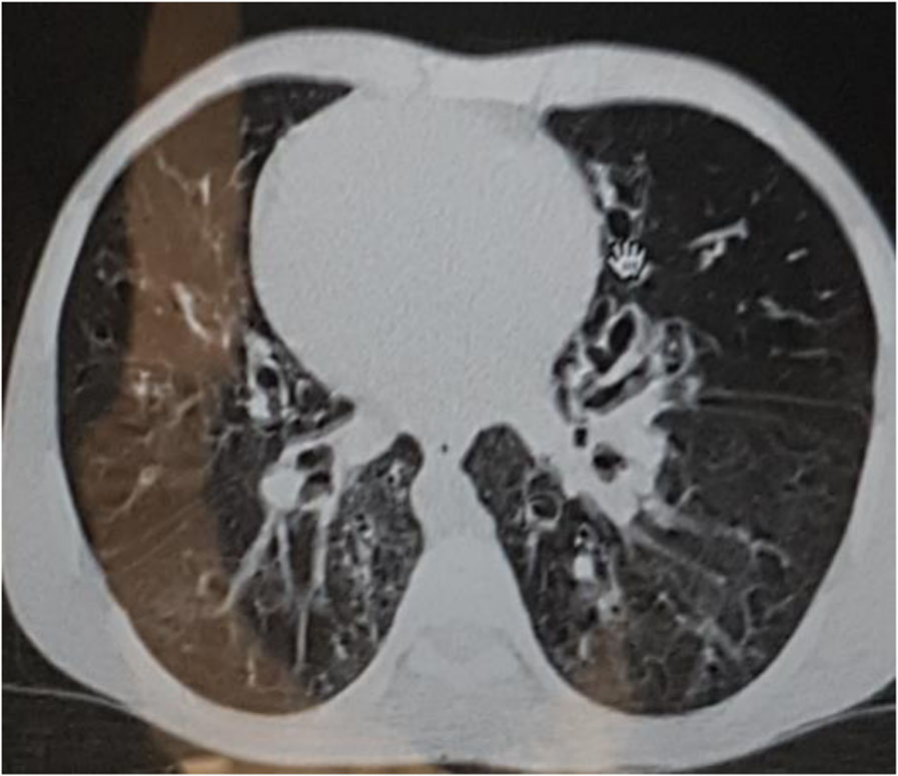
Axial post-contrast computed tomography showing cystic bronchiectasis on both lungs

**Fig. 3:**
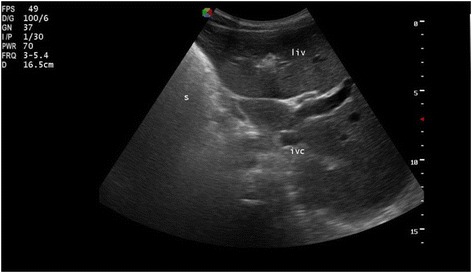
Abdominal ultrasound showing situs inversus (liver and inferior vena cava on left side, and spleen on right side)

A laboratory examination revealed hemoglobin 18 gm/dl (normal, 12–18 gm/dl), total leukocyte count 12,500/μl (normal, 4000–11,000/μl; granulocyte 74%, lymphocyte 15%), and platelet count 350,000/μl (normal, 150,000–450,000/μl). Sputum for acid-fast bacilli (AFB) staining (three times) was negative for *Mycobacterium tuberculosis*. Serum chemistries were normal. A chest X-ray revealed cardiac apex and aortic arch on right side, and fibrotic bands and bronchiectasis on lower field of left lung (Fig. 1). A chest computed tomography (CT) scan showed bronchiectatic changes prominent on both lower lung fields (Fig. 2). Ultrasound examination of his abdomen showed liver and inferior vena cava on left side, and spleen on right side, suggestive of situs inversus (Fig. 3). Then, a diagnosis of KS was made on the basis of clinical presentation and imaging features (Figs. 1, 2 and 3). He was treated with orally administered antibiotics, mucolytic, and chest physiotherapy. He was symptomatically better with the above therapy, and started on long-term low-dose prophylactic antibiotic. He was then referred to the medical chest clinic of our hospital for follow-up.

## Discussion

KS is a rare, autosomal recessive ciliopathic disorder characterized by the clinical triad of chronic sinusitis, bronchiectasis, and situs inversus. Its estimated incidence is approximately 1 in 30,000 live births [1, 2].

Normal ciliary function is critical for respiratory tract host defense, sperm motility, and normal visceral orientation during embryogenesis.

Lack or dysfunction of dynein arms, radial spokes, and microtubules of cilia are recognized structural and functional abnormalities of ciliary ultrastructures, encoded by the mutated genes *DNAI1* and *DNAH5*. These faulty genes cause the cilia to be the wrong size or shape or move in the wrong way, making ciliary motility defective [5, 6].

Abnormal ciliary motility at sites leads to chronic recurrent sinopulmonary infections and infertility. Impaired ciliary motility during embryogenesis predisposes to left–right laterality defects like situs solitus (that is, dextrocardia only) or situs inversus totalis where transpositions of thoracic and abdominal organs are noticed [5–7].

The diagnostic criteria recommended for this syndrome include history of chronic bronchial infection and rhinitis from early childhood, combined with one or more of following features: (a) situs inversus or dextrocardia in a patient or a sibling, (b) alive but immotile spermatozoa, (c) absent or impaired tracheobronchial clearance, and (d) cilia showing characteristic ultrastructural defect on electron microscopy [7, 8].

Laboratory screening tests include exhaled nasal nitric oxide level determination and saccharin test for assessing nasal epithelial mucociliary function. High-speed video microscopy for assessing ciliary beat frequency and pattern, transmission electron microscopic for detecting ultrastructural ciliary defect, and genetic testing for *DNAI1* and *DNAH5* mutations are confirmatory laboratory tests. Abnormal laboratory findings in KS include reduced nasal nitric oxide level (~10% of normal), prolonged saccharin clearance time (>1 hour), reduced ciliary beat frequency (<11 Hz/second), absent ciliary ultrastructure (dynein arms), and mutated *DNAI1* and *DNAH5* genes [6, 7].

Our patient presented with recurrent episodes of sinopulmonary infections. Imaging findings revealed bronchiectasis, dextrocardia, and situs inversus, which met the diagnostic criteria for KS. Laboratory screening and confirmatory tests, which required a better clinical setup, were not done.

As there is no easy, reliable non-invasive diagnostic test for KS and the correct diagnosis is often delayed by years, it may cause chronic respiratory problems with reduced quality of life [7, 9, 10].

Standard treatment for sinopulmonary problems in people with KS includes chest physiotherapy, mucolytics, and antibiotics. A long-term low-dose prophylactic antibiotic is required in those with frequent exacerbation of bronchiectasis (≥3 times/year). Influenza and pneumococcal vaccination should be routinely given [7, 9, 10].

## Conclusions

Patients with KS exist in Ethiopia as cases of chronic recurrent sinopulmonary infections. As there is no easy, reliable non-invasive diagnostic test for KS and the correct diagnosis is often delayed by years, it may cause chronic respiratory problems with reduced quality of life. Genetic counseling and fertility issues should be addressed once KS is diagnosed.

## Abbreviations

AFB: Acid-fast bacilli
BP: Blood pressure
CT: Computed tomography
ENT: Ear, nose, and throat
KS: Kartagener’s syndrome
PR: Pulse rate
RR: Respiratory rate
SaO_2_: Arterial oxygen saturation
T°: Temperature
